# Circulating tumour cells are associated with histopathological growth patterns of colorectal cancer liver metastases

**DOI:** 10.1007/s10585-022-10191-6

**Published:** 2022-11-03

**Authors:** Y. M. Meyer, S. M. Wilting, J. Kraan, P. Olthof, P. Vermeulen, J. Martens, D. J. Grünhagen, S. Sleijfer, C. Verhoef

**Affiliations:** 1 Department of Surgical Oncology and Gastrointestinal Surgery, Erasmus MC Cancer Institute, Rotterdam, The Netherlands; 2 Department of Medical Oncology, Erasmus MC Cancer Institute, Rotterdam, The Netherlands; 3 Translational Cancer Research Unit (GZA Hospitals and University of Antwerp), Antwerp, Belgium

**Keywords:** Circulating tumour cells, Histopathological growth patterns, Colorectal liver metastases, Prediction

## Abstract

Histopathological Growth Patterns (HGPs) have prognostic and predictive value in patients with Colorectal Liver Metastases (CRLM). This study examined whether preoperative measurement of Circulating Tumour Cells (CTCs) is associated with HGP. CTCs were prospectively enumerated in 7.5 ml of blood using the FDA-approved CellSearch system in patients who underwent local treatment of CRLM with curative intent between 2008 and 2021. All CTC samples were collected on the day of local treatment. Patients treated with neoadjuvant chemotherapy for CRLM or with extrahepatic disease at the time of CTC sampling were excluded. HGP was scored retrospectively following the current consensus guidelines. The association between CTCs and HGP was investigated through multivariable logistic regression. Data were available for 177 patients, desmoplastic HGP (dHGP) was observed in 34 patients (19%). There were no statistically significant differences in patient and tumour characteristics between dHGP and non-dHGP at baseline. Patients with dHGP had longer overall – and disease-free survival (logrank p = 0.003 and 0.003, respectively) compared to patients with non-dHGP. CTCs were not detected in 25(74%) of dHGP patients and in 68(48%) of non-dHGP patients (chi-squared p = 0.006). Preoperative absence of CTCs was the only significant predictor for dHGP in multivariable logistic regression (Odds Ratio 2.7, 95%CI 1.1–6.8, p = 0.028), Table 3. Preoperative absence of CTCs is associated with dHGP in chemo naive CRLM patients without extrahepatic disease. Based on our results, CTC count alone is not sufficient to preoperatively identify HGPs, but integration of CTC count in multivariable prediction models may aid the preoperative identification of HGPs of CRLM.

**Supplementary Information:**

The online version contains supplementary material available at 10.1007/s10585-022-10191-6.

## Introduction

Histopathological growth patterns (HGPs) are a prognostic biomarker in patients who have undergone a resection of colorectal cancer liver metastases (CRLM) [1–3]. The evaluation of HGPs is standardized in international consensus guidelines [1, 4]. The assessment of HGPs is reliable, accurate and is conducted on routine Hematoxylin & Eosin (H&E) slides of resected CRLM that are already available for all patients who have undergone resection for CRLM [5]. HGPs can be classified into biologically distinct subtypes: desmoplastic HGP (dHGP) and non-desmoplastic HGP (non-dHGP) [4, 6]. Patients with pure dHGP have favourable Overall- (OS) and Disease Free Survival (DFS) compared to patients with any presence of non-dHGP in the resected tumor [2–4]. The prognostic value of HGPs is independent of other known prognostic factors like Fong clinical risk score [7] and KRAS mutation status [2, 3]. Aside from the prognostic value, recent studies suggest that HGPs are associated with the effectiveness of adjuvant systemic chemotherapy in patients with CRLM [8, 9].

Preoperative knowledge of HGP could provide useful information to guide individual treatment plans, for example to select patients for neoadjuvant therapy. However, preoperative assessment of HGPs is not possible and postoperative pathologic examination of resected metastases is currently the only method to assess HGP of liver metastases [4].

Prediction of HGP through a surrogate marker such as Circulating Tumour Cells (CTCs) may be helpful to integrate HGP in preoperative decision making without the need for upfront pathologic examination. CTCs are a prognostic biomarker in various malignant tumours. It remains unclear whether the presence of CTCs is associated with poor OS and DFS in patients with CRLM [10–13].

The aim of this study was to investigate whether measurement of preoperative CTCs could predict HGPs of resected CRLM.

## Methods

### Study design and patients

A retrospective single center analysis was conducted to explore the association between CTCs and HGP. To include as many patients as possible in this study, patients were selected from two datasets from previous prospective studies on CTCs in patients with CRLM. All patients have undergone curative intent local treatment (i.e. all preoperatively identified lesions were treated) at the Erasmus Medical Center, Rotterdam, the Netherlands between January 2008 and December 2021. All patients had a pathologically confirmed primary colorectal tumour. An overview of both datasets is provided in supplementary table 1. All patients from dataset 1 underwent venous blood sampling and all patients from dataset 2 underwent arterial blood sampling.

Patients were excluded if they had undergone neo-adjuvant chemotherapy for CRLM since neoadjuvant chemotherapy may influence HGP assessment and CTC enumeration [14–16]. Patients were also excluded if extrahepatic disease was present at the time of CTC sampling.

### Assessments

Per patient 30 mL blood was collected in CellSave tubes and assessed for CTCs at a local Erasmus MC laboratory. All study samples were collected preoperatively on the day of local treatment of CRLM. Samples were processed within 24 h of collection. The 30 mL whole blood samples were reduced to 7.5 mL enriched blood samples by ficoll density gradient separation, which has been described previously [17, 18]. CTCs were enumerated using the CELLSEARCH CTC kit (Menarini Silicon Biosystems, Castel Maggiore, Italy). CTC enumeration results were reviewed by 2 trained operators [13].

The CELLSEARCH CTC kit contains magnetic beads coated with anti-epithelial-cell adhesion molecule antibodies in order to imunomagnetically enrich epithelial cells from whole blood. The remaning cells are stained with DAPI, anti CK 8, 18 or 19 antibodies and anti CD45 antibodies. The sample is then transferred to a Magnest Cell Preservation Device, after which the cells are scanned by the Cell Spotter Analyzer, which is a four-color semi-automated fluorescence microscope. The images are assessed by trained readers. CTCs are selected based on the following criteria: size ≥ 4 µm, round to oval morphology, positive staining for CK8, 18 or 19, a visible DAPI positive nucleus, at least 50% overlap between nucleus and cytoplasm and negative staining for CD45.

HGPs were scored retrospectively on H&E slides of liver metastases following the current consensus guidelines [1, 4]. HGPs were scored per H&E slide as a percentage of the total tumour-liver interface on all available H&E slides for each patient. The average HGP was subsequently calculated per lesion and (in case of multiple lesions) per patient to yield a single HGP score for each patient [1, 4]. Based on recent studies, the cutoff for dHGP was set at pure dHGP (100%) versus any amount of non-dHGP [2–4]. Pure dHGP will be referred to as “dHGP” and any amount of non-dHGP will be referred to as “non-dHGP” for the remainder of this paper.

Fong clinical risk score was calculated for all patients and classified as low (< 3 points) and high risk (≥ 3 points) [7].

### Statistics

Baseline characteristics for dHGP and non-dHGP, as well as for the two datasets were compared using fischer’s exact test for discrete variables and the Kruskal–Wallis test for continuous variables. Continuous variables are represented as median (95% CI) unless indicated otherwise. OS and DFS of the HGPs and the OS and DFS of detectable and non-detectable CTCs were assessed via Kaplan–Meier analysis and compared using the log-rank test. OS was defined as the time in months from resection of CRLM to death of any cause. DFS was defined as the time in months from resection of CRLM to recurrence of disease or death. The prognostic value of CTCs was evaluated through multivariable cox regression analysis. The association between CTCs and HGP was evaluated through multivariable logistic regression. Patient and tumour characteristics were evaluated through univariable logistic regression. Predictors with a p < 0.2 were included in multivariable logistic regression and subsequently excluded via backward elimination. Number of CRLM was included in multivariable analysis to correct for tumour load. A p-value of < 0.05 was considered statistically significant.

## Results

A total of 177 patients were included who underwent surgery between January 2008 and December 2021. The baseline characteristics are shown in Table 1. No statistically significant differences in patient and tumour characteristics were observed between dHGP and non-dHGP patients at baseline. There were 34 patients with dHGP (19%). Five-year OS in the dHGP patients was 86% (95% CI 73–100%) compared to 44% (95%CI 35–56%) in non-dHGP patients (logrank p = 0.003). Five-year DFS was 39% (95% CI 23–65%) in the dHGP patients compared to 19% (95% CI 13%-28%) in non-dHGP patients, p = 0.003.

**Table 1:** Baseline characteristics per HGP

n		dHGP	non-dHGP	p	Missing
		34	143		%
CTC count (median [IQR])		0.0 [0.0, 0.8]	1.0 [0.0, 2.0]	**0.034**	0
CTC (%)	Detectable	9 (26)	75 (52)	**0.006**	0
	Not detectable	25 (74)	68 (48)		
CTC sampling	Venous	13 (38)	73 (51)	0.179	0
	Arterial	21 (62)	70 (49)		
Sex (%)	Male	22 (65)	95 (66)	0.848	0
Age (years, median [IQR])		66.7 [60.6, 71.7]	67.1 [60.3, 73.8]	0.697	0
ASA class (%)	ASA Class I	6 (18)	36 (25)	0.453	0
	ASA Class II	22 (65)	91 (64)		
	ASA Class III	6 (18)	16 (11)		
Location Primary tumour (%)*	Right-sided	8 (24)	32 (22)	0.863	3
	Left-sided	15 (44)	58 (41)		
	Rectum	10 (29)	49 (34)		
Resection approach (%)*	Primary first	31 (91)	129 (90)	0.863	0
	Synchronous	3 (9)	14 (10)		
T- stage (%)*	T1	1 (3)	4 (3)	0.78	1
	T2	7 (21)	20 (14)		
	T3	22 (65)	103 (72)		
	T4	4 (12)	14 (10)		
N- stage (%)	N +	15 (44)	85 (59)	0.105	0
Synchronous metastases^1^ (%)	Synchronous	31 (36)	25 (27)	0.22	0
Disease Free Interval^2^ (%)	> 1 year	27 (31)	43 (47)	**0.031**	0
	= / < 1 year	59 (69)	48 (53)		
Number of Liver metastases (%)	= / < 1	38 (44)	53 (58)	0.061	0
	> 1	48 (56)	38 (42)		
Preoperative CEA (ug/l) (%)*	= / < 200	31 (91)	125 (88)	0.225	9
	> 200	0 (0)	6 (4)		
Diameter of largest liver metastasis (cm) (%)	= / < 5	32 (94)	121 (85)	0.146	0
	> 5	2 (6)	22 (15)		
FONG score (%)	Low	55 (64)	69 (76)	0.085	0
	High	31 (36)	22 (24)		
Bilobar liver metastases (%)	Unilobar	60 (70)	69 (76)	0.365	0
	Bilobar	26 (30)	22 (24)		
Resection radicality (%)	R0	79 (92)	81 (89)	0.52	0
	R1	7 (8)	10 (11)		
Five-year overall survival %		86 (73–100)	44 (35–56)	**0.003**	2.2
Five-year disease free survival %		39 (23–65)	19 (13–28)	**0.003**	17.5

^1^Synchronous metastases defined as liver metastases detected ≤ 3 months after resection of the primary tumour

^2^Disease Free interval = time resection of primary tumour and detection of liver metastases

*Percentages do not add up to 100%, due to missing date

p-values are bold for statistically significant differences

**Fig. 1:**
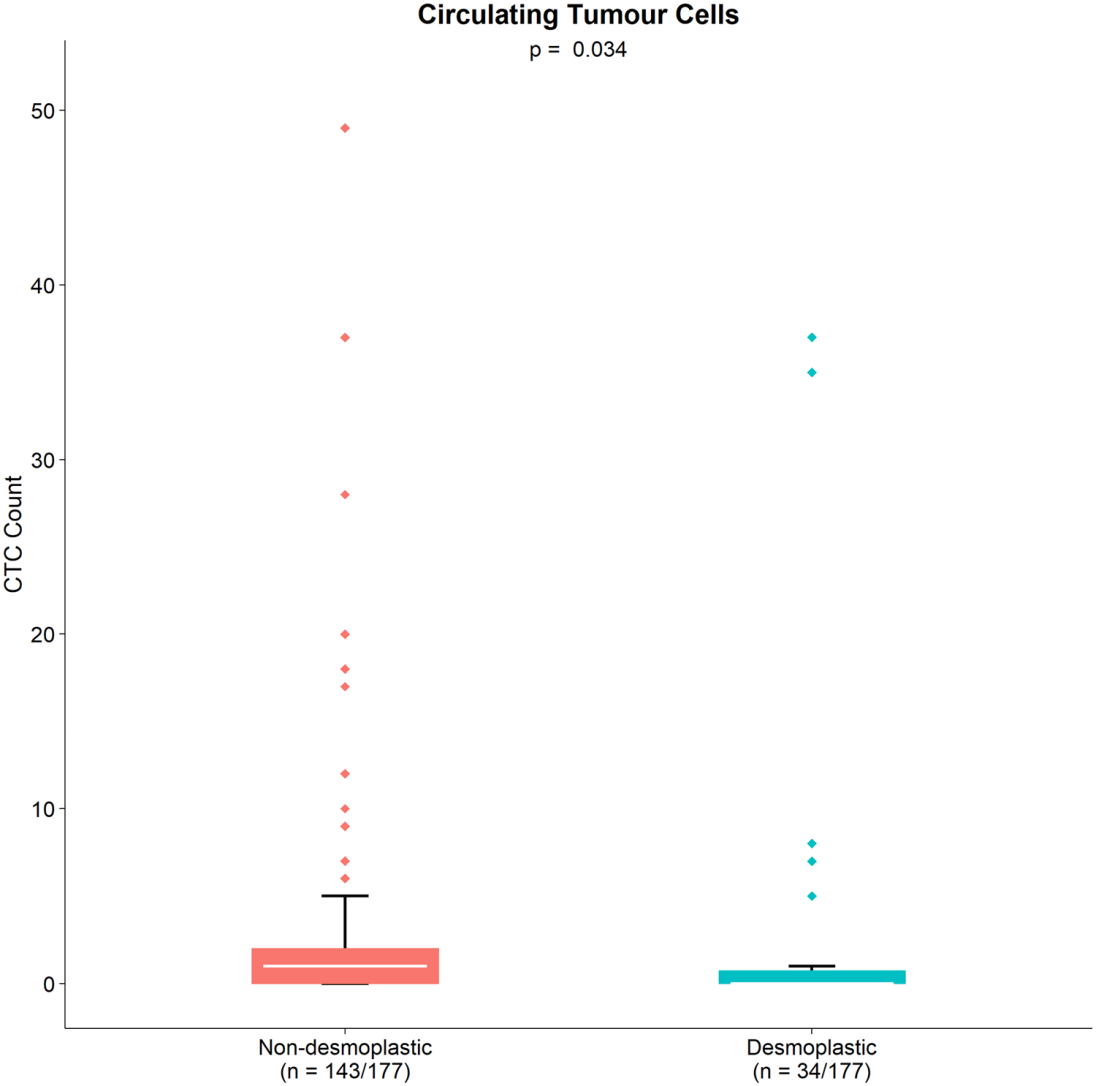
CTC counts per HGP group

There was a statistically significant difference in CTC counts between the HGP groups. The median CTC count for dHGP was 0.0 (IQR0.0–0.8), the median CTC count for non-dHGP was 1.0 (IQR 0.0–2.0), p = 0.034. The distribution of the CTC counts per HGP groups is shown in Fig. 1.

Table 2 shows the baseline characteristics per CTC category. The group with detectable CTCs contained more male patients than the group without detectable CTCs, 68 (73%) compared to 49 (58%), p = 0.038. There were more patients with metastasis > 5 cm in diameter in the detectable CTC group than in the group without detectable CTCs, 20 patients (24%) versus 4 patients (4%) respectively, p < 0.001. No other statistically significant differences were observed between these groups. Five-year OS was 39% (95% CI 30–50%) for patients with detectable CTCs and 57% (95%CI 49–66%) for patients without detectable CTCs (p = 0.065). DFS was 23% (95% CI 16–34%) for detectable CTCs and 27% (95% CI 20–36%) for non-detectable CTCs, p = 0.879. CTC status (detectable/non-detectable) was not associated with OS (HR 0.8, 95% CI 0.5–1.3, p = 0.35) or DFS (HR 95%CI 1.0, 0.7–1.5, p = 0.84) in multivariable analysis. The multivariable analysis corrected for the following known predictors of survival in patients with CRLM: HGP, location of the primary colorectal tumour, lymph node status of the primary tumour, disease free interval between resection of the primary tumour and detection of CRLM, number of CRLM, diameter of the largest CRLM, CEA before liver resection. Significant predictors for OS in multivariable analysis were: dHGP (HR 0.45, 95% CI 0.22–0.89, p = 0.02), left-sided primary tumour (HR 0.47, 95%CI 0.24–0.92, p = 0.03) and diameter of the largest CRLM > 5 cm (HR 2.41, 95%CI 1.16–5.02. p = 0.02). Significant predictors for DFS in multivariable analysis were: dHGP (HR 0.45 95%CI 0.26–0.77), Disease Free Interval < 12 months (HR 1.74 95%CI 1.15–2.36, p < 0.01) and > 1 CRLM (HR 1.83 95%CI 1.22–2.74, p < 0.01). Kaplan–Meier curves as well as the full Cox regression analysis are provided as supplementary Fig. 1 and supplementary table 2, respectively.

**Table 2:** Baseline Characteristics per CTC category

n		Detectable	Not detectable	p	Missing
		84	93		%
HGP(%)	dHGP	9 (11)	25 (27)	**0.006**	0
	non-dHGP	75 (89)	68 (73)		
CTC count (median [IQR])		2.0 [1.0, 5.0]	0.0 [0.0, 0.0]	-	0
CTC sampling	Venous	43 (51)	43 (46)	0.51	0
	Arterial	41 (49)	50 (54)		
Sex (%)	Male	49 (58)	68 (73)	**0.038**	0
Age (median [IQR])		67.4 [61.8, 73.0]	65.8 [58.4, 73.8]	0.19	0
ASA class (%)	ASA Class I	19 (23)	23 (25)	0.912	0
	ASA Class II	55 (65)	58 (62)		
	ASA Class III	10 (12)	12 (13)		
Location primary tumour (%)	Right-sided	20 (24)	21 (23)	0.888	0
	Left-sided	34 (40)	41 (44)		
	Rectum	30 (36)	31 (33)		
Resection approach (%)	Primary first	76 (90)	84 (90)	0.972	0
	Synchronous	8 (10)	9 (10)		
T- stage (%)*	T1	2 (2)	3 (3)	0.163	1
	T2	8 (10)	19 (20)		
	T3	62 (74)	63 (68)		
	T4	11 (13)	7 (8)		
N- stage (%)	N +	53 (63)	47 (51)	0.092	0
Synchronous metastases^1^ (%)	Synchronous	21 (25)	35 (38)	0.071	0
DFI^2^ (%)	> 1 year	36 (43)	34 (37)	0.392	0
	= / < 1 year	48 (57)	59 (63)		
Number of liver metastases (%)	= / < 1	46 (55)	45 (48)	0.397	0
	> 1	38 (45)	48 (52)		
Preoperative CEA(%)*	= / < 200	76 (90)	80 (86)	0.096	9
	> 200	5 (6)	1 (1)		
Diameter of largest liver metastasis (%)	= / < 5	64 (76)	89 (96)	** < 0.001**	0
	> 5	20 (24)	4 (4)		
FONG score (%)	Low	54 (64)	70 (75)	0.111	0
	High	30 (36)	23 (25)		
Bilobar liver metastases (%)	Unilobar	66 (79)	63 (68)	0.106	0
	Bilobar	18 (21)	30 (32)		
Resection radicality (%)	R0	76 (90)	84 (90)	0.972	0
	R1	8 (10)	9 (10)		
5 year overall survival (%)		39 (30–50)	57 (49–66)	0.065	2
5 year disease free survival (%)		23 (16–34)	27 (20–36)	0.879	18
Recurrence					
Intrahepatic		36 (43)	32 (34)	0.525	33
Extrahepatic		37 (45)	33 (35)	0.525	33

^1^Synchronous metastases defined as liver metastases detected ≤ 3 months after resection of the primary tumour

^2^Disease Free interval = time resection of primary tumour and detection of liver metastases

*Percentages do not add up to 100%, due to missing data

p-values are bold for statistically significant differences

CTCs were not detected in 74% (n = 25) of patients with dHGP and in 48% of patients (n = 68) with non-dHGP (p = 0.006). There were no statistically significant differences in these proportions between arterial and venous blood samples. An overview of the CTC counts is provided in supplementary table 3.

The sensitivity of absent CTCs for dHGP was 74%, the specificity of absent CTCs for dHGP was 58%. The positive predictive value of absent CTCs for dHGP was 27%, the negative predictive value of absent CTCs for dHGP was 89%.

The absence of CTCs remained the only significant predictor for dHGP in multivariable logistic regression with an odds ratio 2.7 (95%CI 1.1–6.8; p = 0.028). The uni-and multivariable results are shown in Table 3.

**Table 3:** Uni- and multivariable logistic regression predicting dHGP

	Univariable	p	Multivariable	p
CTC not detectable	3.1 [1.4–7.4]	**0.008**	2.7 [1.1–6.8]	**0.028**
Primary tumour				
Left sided	Reference	–	–	–
Right sided	1.0 [0.4–2.8]	0.945	–	–
Rectum	0.8 [0.2–2.3]	0.700	–	–
Lymph node positive primary	0.5 [0.2–1.2]	0.113	0.6 [0.2–1.3]	0.182
DFI < 1 year	0.9 [0.4–2.0]	0.829	–	–
Number of CRLM > 1	0.7 [0.3–1.5]	0.338	0.5 [0.2–1.1]	0.105
Diameter of largest CRLM > 5 cm	0.2 [0.0–1.2]	0.174	0.5 [0.0–3.2]	0.541
Preoperative CEA	1.0 [0.9–1.0]	0.05	1.0 [0.9–1.0]	0.05

*CTC* Circulating Tumour Cells, *DFI* Disease Free Interval between resection of the primary tumour and detection of Liver metastases, *CRLM* ColoRectal Liver Metastases

## Discussion

In this retrospective study including 177 patients who underwent liver resection for CRLM an association was found between HGPs and CTCs. In multivariable logistic regression, the absence of CTCs was associated with presence of dHGP. However, CTCs were absent in 74% of patients with dHGP compared to 48% of non-dHGP. Since non-dHGP is more common than dHGP, the measurement of CTCs alone is therefore not sufficient to predict HGP preoperatively to use it in a clinical setting. Given the association with HGP, CTCs may be useful as a factor in multivariable preoperative prediction models for HGP. There are several potential factors that may be predictive for HGP. Recent studies suggest that the histopathology of the primary colorectal tumour may be correlated to HGPs of CRLM [19, 20]. Studies have also shown promising results in predicting HGP based on preoperative imaging using radiomics and artificial intelligence [21, 22]. Combining the predictors above could result in a model that may be accurate enough to use in preoperative decision making.

HGPs are a promising biomarker in patients with CRLM, which has several potential advantages for clinical use. The evaluation of HGPs is standardized in international consensus guidelines [1, 4]. The assessment of HGPs is reliable, accurate and is conducted on routine H&E slides of resected CRLM that are available for all patients who have undergone resection for CRLM [5]. HGPs are a strong predictor of OS and DFS after curative intent resection of CRLM and are independent of other known prognostic factors like Fong clinical risk score[7] and KRAS mutation status [2, 3, 23]. In addition, there is evidence to suggest that HGPs may also have predictive value in patients with CRLM [8, 9]. A disadvantage of HGPs is that they can only be scored postoperatively on slides of resected CRLM, making them unavailable until resection has taken place. Preoperative assessment of HGP would allow clinicians to fully utilize the prognostic and predictive capabilities of HGP [8]. Prediction of HGPs using a minimally invasive method would enable preoperative the use of HGPs.

CTCs may be a prognostic marker for OS and DFS in patients with CRLM [10, 24, 25]. The presence of CTCs has been associated with worse outcomes, although the most informative cutoff remains unclear [26]. CTCs may also be predictive in patients who receive chemotherapy for metastatic colorectal cancer [27]. To our knowledge, this is the first study investigating the association between the phenotype of CRLM and CTCs.

No association between CTC counts and OS or DFS was found in the current study. However, no conclusions on this subject can be drawn from this data as the retrospective study design, with different methods of CTC sampling does not lend itself well to answer this research question. The study may not have the statistical power to detect clinically relevant differences in OS or DFS between different CTC groups with statistical significance. A prospective, multicenter Dutch study evaluating the association between CTCs and DFS has almost completed the follow-up [28].

The difference in the proportion of patients with non-detectable CTCs between dHGP and non-dHGP patients in this study has yet to be explained. Previous studies have shown an association between tumour burden and CTC counts in patients with CRLM [29]. In the current study the group with detectable CTCs had significantly larger metastases. There was no significant difference in tumour burden between dHGP and non-dHGP, there was even a trend towards more liver metastases in dHGP compared to non-dHGP. Moreover, a statistically significant association remained between HGPs and CTCs in multivariable analysis when correcting for diameter and number of liver metastases. Tumour burden appears to offer no explanation for the differences in CTC status between dHGP and non-dHGP found in this study. There is evidence that patients with non-dHGP have a higher risk of extrahepatic- and multi-organ recurrence after treatment of CRLM [30]. The higher proportion of patients with extrahepatic disease and the higher proportion of detectable CTCs in patients with non-dHGP compared to dHGP may be the result of a shared underlying mechanism. Previous studies on the tumour microenvironment of the different HGPs in CRLM have shown an increased immune infiltrate, enriched with cytotoxic CD8 + T-cells in dHGP when compared to non-dHGP [31]. The association between immune infiltrate including CD8 + T-cells with prognosis has been shown for both primary and metastatic colorectal cancer [32–36] and suggests more anti-tumour immune activity in dHGP, which may contribute to the favourable OS and DFS for dHGP compared to non-dHGP [2, 3, 31]. Similarly, an effective immune response may affect CTC counts, where evasion of immune surveillance is proposed as one of the major contributors to the presence of CTCs in the circulation [37, 38]. In summary, liver metastases with pure dHGP are associated with an increased immune response in the liver[31] and the associated lack of CTCs signifies anti-tumour immunity in dHGP. However, the significance of our finding needs to be validated after which causality can be explored.

A limitation of the study is the combination of two separate datasets for the analysis. An important difference between the two datasets that were used is that the CTCs were enumerated in venous blood in the 86 patients of dataset 1 and in arterial blood in the 91 patients of dataset 2. Previous studies have suggested that arterial blood samples may be superior to detect CTCs compared to venous samples, [39, 40] even though arterial samples may be more challenging to collect in clinical practice. In the current study, we found no statistically significant difference in median CTC count between both datasets. In addition, the percentages of detectable CTCs between dHGP and non-dHGP for both sampling methods were similar. Given the similarities in CTC counts, HGP proportions, and overall patient and tumour characteristics between both cohorts, it is unlikely that the use of two cohorts has compromised the current study.

Another limitation of this study is external validity. To relate HGP of liver metastases to CTCs, patients with extrahepatic disease were excluded to prevent measurement of CTCs from other metastatic locations than CRLM. Patients receiving neo-adjuvant chemotherapy were excluded as well, because previous studies have shown that neo-adjuvant chemotherapy may alter the HGPs of CRLM [14], and neo-adjuvant chemotherapy may also influence the detection of CTCs [15, 16]. This selection may be appropriate for the current research question, but the strict in- and exclusion criteria result in a population that may not fully resemble the current clinical practice. For instance, most patients in this study had favorable tumour characteristics and a low tumour load. The majority (70%) had a low Fong Clinical Risk score [7].

The lack of genetic mutation data is another limitation of this study. KRAS, BRAF and MSI status is not routinely evaluated in Dutch clinical practice, leading to this data being unavailable for most patients of the cohort. Primary tumour location was used as a covariate in multivariable analysis, which may somewhat mitigate the lack of mutation data as right sided primary tumours are correlated with increased incidence of genetic mutations [41].

In conclusion, in this study the absence of preoperative CTC was associated with dHGP in patients with colorectal liver metastases, however, CTCs alone are insufficient for preoperative prediction of HGP for use in clinical practice. Based on our results CTC enumeration could represent a valuable addition to preoperative prediction models for HGP.

## Supplementary Information

Below is the link to the electronic supplementary material.

Supplementary file1 (DOCX 79 KB)

## Data Availability

The datasets generated during and/or analysed during the current study are available from the corresponding author on reasonable request.
