# Supplementary material for: Circulating tumour cells are associated with histopathological growth patterns of colorectal cancer liver metastases
Source: Clin Exp Metastasis. 2022 Nov 3;40(1):69–77. doi: 10.1007/s10585-022-10191-6 (PMC9898367; doi:10.1007/s10585-022-10191-6)

# Supplementary

Supplementary table 1. Overview of the baseline characteristics per dataset

|  |  | Dataset 1 | Dataset 2 | p | Missing |
| --- | --- | --- | --- | --- | --- |
| n |  | 86 | 91 |  | % |
| HGP(%) | dHGP | 13 (15) | 21 (23) | 0.179 |  |
|  | non-dHGP | 73 (85) | 70 (77) |  |  |
| CTC count (median [IQR]) |  | 0.5 [0.0, 2.0] | 0.0 [0.0, 1.5] | 0.497 | 0 |
| CTC (%) | Detectable | 43 (50) | 41 (45) | 0.51 | 0 |
|  | Not detectable | 43 (50) | 50 (55) |  |  |
| Sex (%) | Male | 56 (65) | 61 (67) | 0.788 | 0 |
| Age (median [IQR]) |  | 68.8 [61.7, 74.1] | 65.6 [58.4, 72.4] | 0.152 | 0 |
| ASA class (%) | ASA Class I | 8 (9) | 34 (37) | **<0.001** | 0 |
|  | ASA Class II | 63 (73) | 50 (55) |  |  |
|  | ASA Class III | 15 (17) | 7 (8) |  |  |
| Location Primary tumour (%) | Right-sided | 25 (29) | 16 (18) | **0.035** | 0 |
|  | Left-sided | 39 (45) | 36 (40) |  |  |
|  | Rectum | 22 (26) | 39 (43) |  |  |
| Resection approach (%) | Primary first | 80 (93) | 80 (88) | 0.249 | 0 |
|  | Synchronous | 6 (7) | 11 (12) |  |  |
| T- stage (%)* | T1 | 3 (3) | 2 (2) | **0.02** | 1 |
|  | T2 | 12 (14) | 15 (16) |  |  |
|  | T3 | 56 (65) | 69 (76) |  |  |
|  | T4 | 15 (17) | 3 (3) |  |  |
| N- stage (%) | N+ | 53 (62) | 47 (52) | 0.181 | 0 |
| Synchronous metastases (%) | Synchronous | 31 (36) | 25 (27) | 0.22 | 0 |
| DFI (%) | >1 year | 27 (31) | 43 (47) | 0.031 | 0 |
|  | =/<1 year | 59 (69) | 48 (53) |  |  |
| Number of Liver metastases (%) | =/<1 | 38 (44) | 53 (58) | 0.061 | 0 |
|  | >1 | 48 (56) | 38 (42) |  |  |
| Preoperative CEA(%)* | =/<200 | 75 (87) | 81 (89) | 0.926 | 9 |
|  | >200 | 3 (3) | 3 (3) |  |  |
| Diameter of largest liver metastasis (%)* | =/<5 | 72 (84) | 81 (89) | 0.304 | 1 |
|  | >5 | 14 (16) | 10 (11) |  |  |
| FONG score (%) | Low | 55 (64) | 69 (76) | 0.085 | 0 |
|  | High | 31 (36) | 22 (24) |  |  |
| Bilobar liver metastases (%) | Unilobar | 60 (70) | 69 (76) | 0.365 | 0 |
|  | Bilobar | 26 (30) | 22 (24) |  |  |
| Resection radicality (%) | R0 | 79 (92) | 81 (89) | 0.52 | 0 |
|  | R1 | 7 (8) | 10 (11) |  |  |

CTC= Circulating Tumour Cells, DFI= Disease Free Interval between resection of the primary tumour and detection of Liver metastases, CRLM= ColoRectal Liver Metastases. * Percentages do not add up to 100% due to missing data.

Supplementary table 2. Uni- and multivariable Cox regression for Overall and Disease-free Survival

| **Overall Survival** |  |  |  |  |
| --- | --- | --- | --- | --- |
|  | **Univariable** | **p** | **Multivariable** | **p** |
| CTCs not-detectable | 0.66 [0.43-1.03] | 0.07 | 0.87 [0.53-1.42] | 0.57 |
| dHGP | 0.40 [0.21-0.75] | **<0.01** | 0.45 [0.22-0.89] | **0.02** |
| Location Primary tumour |  |  |  |  |
| Right-sided | Reference | - | Reference | - |
| Left-sided | 0.47 [0.25-0.86] | **0.01** | 0.47 [0.24-0.92] | **0.03** |
| Rectum | 0.89 [0.51-1.57] | 0.7 | 1.07 [0.59-1.95] | 0.83 |
| Node positive primary | 1.75 [1.12-2.73] | 0.01 | 1.56 [0.96-2.52] | 0.07 |
| DFI <12 months | 1.06 [0.68-1.65] | 0.79 | 0.97 [0.59-1.59] | 0.89 |
| >1 CRLM | 1.65 [1.07-2.57] | **0.02** | 1.55 [0.95-2.54] | 0.08 |
| Diameter CRLM >5 cm | 2.28 [1.15-4.52] | **0.02** | 2.41 [1.16-5.02] | **0.02** |
| Preoperative CEA >200 ug/l | 1.17 [0.37-3.71] | 0.78 | 0.69 [0.20-2.29] | 0.54 |
|  |  |  |  |  |
| **Disease Free Survival** |  |  |  |  |
|  | **Univariable** | **p** | **Multivariable** | **p** |
| CTCs not-detectable | 1.03 [0.72-1.46] | 0.88 | 1.17 [0.79-1.74] | 0.43 |
| dHGP | 0.48 [0.29-0.79] | **<0.01** | 0.45 [0.26-0.77] | **<0.01** |
| Location Primary tumour |  |  |  |  |
| Right-sided | Reference | - | Reference | - |
| Left-sided | 0.90 [0.57-1.43] | 0.67 | 0.95 [0.58-1.57] | 0.85 |
| Rectum | 1.03 [0.64-1.64] | 0.91 | 1.17 [0.72-1.91] | 0.53 |
| Node positive primary | 1.46 [1.02-2.09] | **0.04** | 1.40 [0.95-2.06] | 0.09 |
| DFI <12 months | 2.02 [1.38-2.95] | **<0.001** | 1.74 [1.15-2.63] | **<0.01** |
| >1 CRLM | 2.05 [1.43-2.94] | **<0.001** | 1.83 [1.22-2.74] | **<0.01** |
| Diameter CRLM >5 cm | 1.07 [0.59-1.94] | 0.83 | 1.44 [0.76-2.73] | 0.26 |
| Preoperative CEA >200 ug/l | 0.96 [0.35-2.61] | 0.94 | 0.74 [0.26-2.12] | 0.58 |

CTC= Circulating Tumour Cells, DFI= Disease Free Interval between resection of the primary tumour and detection of Liver metastases, CRLM= ColoRectal Liver Metastases.

Supplementary table 3. Circulating Tumour Cell counts

|  |  | **dHGP** | **non-dHGP** | **p** |
| --- | --- | --- | --- | --- |
| **Overall Cohort** |  | **34** | **143** |  |
| CTC (%) | Detectable | 9 (26) | 75 (52) | **0.006** |
|  | Not detectable | 25 (74) | 68 (48) |  |
| **Arterial blood samples** |  | **21** | **70** |  |
| CTC (%) | Detectable | 6 (29) | 35 (50) | 0.083 |
|  | Not detectable | 15 (71) | 35 (50) |  |
| **Venous blood samples** |  | **13** | **73** |  |
| CTC (%) | Detectable | 3 (23) | 40 (55) | **0.035** |
|  | Not detectable | 10 (77) | 33 (45) |  |

CTC= Circulating Tumour Cell, dHGP= Desmoplastic HGP

Supplementary table 4. Mutation data for the HGP and CTC groups

|  |  | **dHGP** | **non-dHGP** | **p** | **Missing (%)** |
| --- | --- | --- | --- | --- | --- |
| n |  | 34 | 143 |  |  |
| **KRAS (%)** | wildtype | 1 (50) | 15 (60) | 0.782 | **84.7** |
|  | mutation | 1 (50) | 10 (40) |  |  |
| **NRAS (%)** | wildtype | 2 (100) | 14 (100) | NA | **91** |
|  | mutation | 0 | 0 |  |  |
| **BRAF (%)** | wildtype | 2 (100) | 24 (96) | 0.773 | **84.7** |
|  | mutation | 0 (0) | 1 (4) |  |  |
| **MSI (%)** | MSS | 9 (82) | 35 (100) | 0.01 | **74** |
|  | MSI | 2 (18) | 0 (0) |  |  |
|  |  | **CTC Detectable** | **CTC Not detectable** | p | **Missing (%)** |
| n |  | 84 | 93 |  |  |
| **KRAS (%)** | wildtype | 7 (58) | 9 (60) | 0.93 | **84.7** |
|  | mutation | 5 (42) | 6 (40) |  |  |
| **NRAS (%)** | wildtype | 10 (100) | 6 (100) | NA | **91** |
|  | mutation | 0 | 0 |  |  |
| **BRAF (%)** | wildtype | 12 (100) | 14 (93) | 0.362 | **84.7** |
|  | mutation | 0 (0) | 1 (7) |  |  |
| **MSI (%)** | MSS | 22 (96) | 22 (96) | 1 | **74** |
|  | MSI | 1 (4) | 1 (4) |  |  |

Supplementary figure 1. Kaplan-Meier Curvers for Overall and Disease-Free Survival
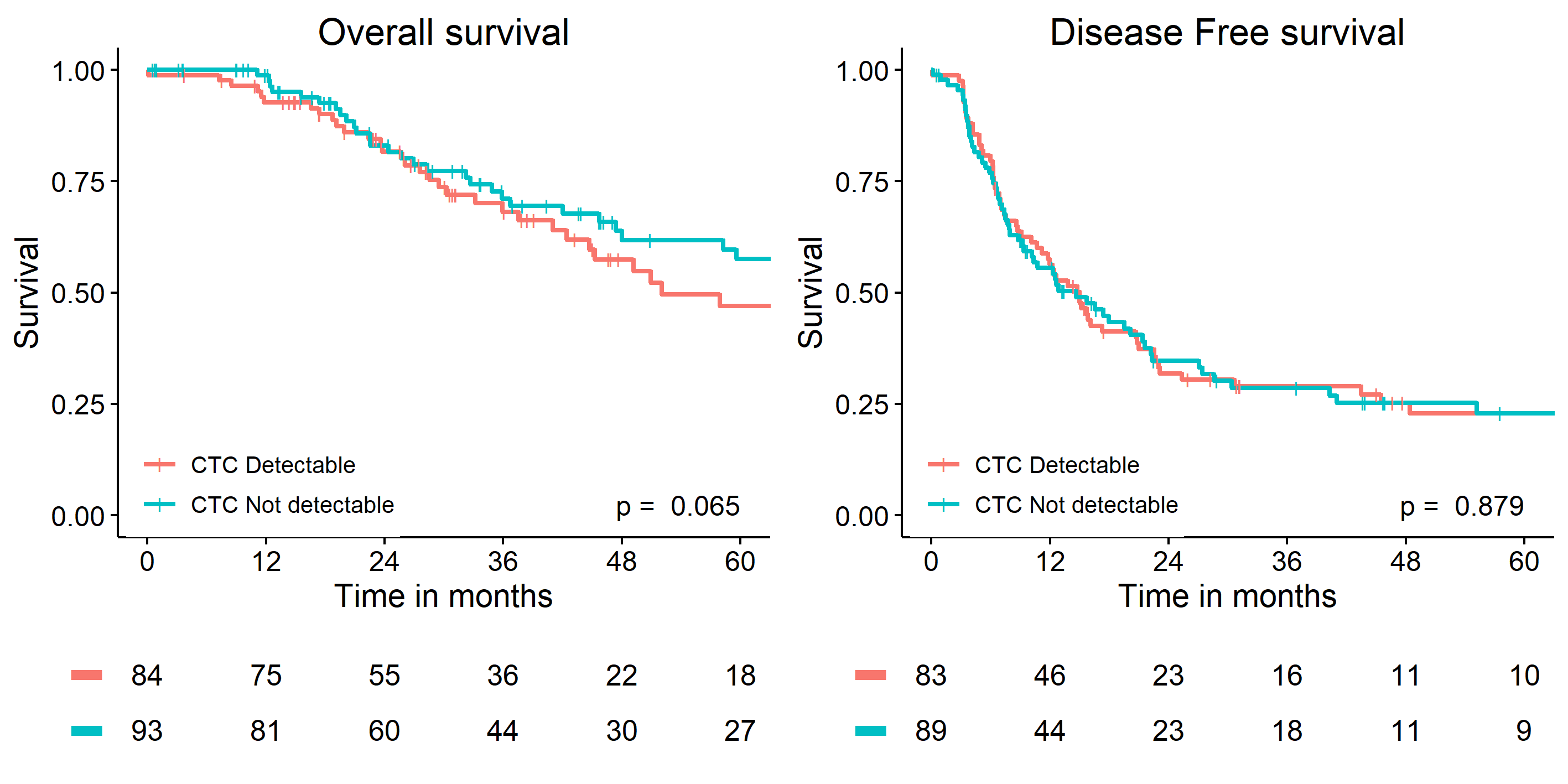

Supplement: Supplementary file 1 — Supplementary file1 (DOCX 79 KB) [file 10585_2022_10191_MOESM1_ESM.docx]
